# When the World Pivots: Changes in Infant Negative Affect Trajectories Following the Onset of the COVID‐19 Pandemic

**DOI:** 10.1111/infa.70041

**Published:** 2025-08-06

**Authors:** Joscelin Rocha‐Hidalgo, Brendan Ostlund, Vanessa LoBue, Kristin A. Buss, Koraly E. Pérez‐Edgar

**Affiliations:** ^1^ Department of Psychology The Pennsylvania State University University Park Pennsylvania USA; ^2^ Department of Psychology Rutgers University – Newark New Brunswick New Jersey USA

**Keywords:** COVID‐19, maternal anxiety, negative affect, neighborhood disadvantage, pandemic

## Abstract

Research on the COVID‐19 pandemic's effect on infant emotional development has produced mixed results, often limited by methodological constraints, such as not having access to data prior to and after pandemic onset. This study helps overcome these limitations by analyzing data from 330 infants (51% female; 54% White, non‐Hispanic) across five points in the first 2 years of life, from October 2016 to August 2021. Multilevel growth models indicated that negative affect decreased following pandemic onset, contrary to the expected and observed increase in negative affect prior to the pandemic. Higher levels of contextual risk (maternal trait anxiety, neighborhood disadvantage) were associated with higher levels of infant negative affect, irrespective of the pandemic. These findings further our understanding of the pandemic's impact on child development.

## 1 Introduction

The World Health Organization declared a global pandemic on March 11, 2020, to stop the spread of a novel infectious coronavirus (SARS‐CoV‐2) that causes coronavirus disease (COVID‐19). In response, governments around the globe instituted unprecedented mitigation measures that closed much of civic life, including schools, offices, and all but essential businesses and services. These closures required a radical shift in the daily lives of individuals and families. For children, this meant little to no in‐person contact with teachers and peers and much greater time within smaller familial units. This constriction of social contact was most acute for younger children, as adolescents often turned to social media and other electronic conduits for social interaction (Metherell et al. 2022; Myruski et al. 2024). Emerging evidence indicates that the onset of the COVID‐19 pandemic and the ensuing social, economic, and health‐related hardships contributed to a rise in mental health concerns for individuals from early childhood through adulthood (Kauhanen et al. 2023; Panchal et al. 2023; Racine et al. 2021, 2025; Werchan et al. 2022; Xiong et al. 2020). However, less is known about the impact of the pandemic on infant emotional development, a group for whom early experience may uniquely shape psychological health for years to come (Padmanabhan et al. 2016).

Here, we built on the emerging data to examine emotional trajectories in the first 2 years of life to further characterize the COVID‐19 pandemic's effect on emotional development in infancy (Buthmann et al. 2022; Fiske et al. 2021; López‐Morales et al. 2022; MacNeill et al. 2023; Roche et al. 2022; Werchan et al. 2022). We did so by leveraging a longitudinal study that, by chance, captured a natural experiment in the timing of the COVID‐19 pandemic onset (Pérez‐Edgar et al. 2021). That is, some infants in this sample completed participation and data collection before the onset of the pandemic, while others were exposed to pandemic‐related disruptions during data collection at varying points up to 2 years of age. As a result, we could compare trajectories in an early risk factor for later psychopathology—negative affect—in a single sample of infants differing only in the timing of the COVID‐19 pandemic's onset.

### 1.1 COVID‐19 Pandemic's Impact on Childhood Mental Health

The emotional toll of COVID‐19 on mental health is evident across the lifespan. Nearly half of children and adolescents reported elevated anxiety symptoms during the pandemic, with rates increasing with age (Panchal et al. 2023), potentially peaking in December 2020 (Metherell et al. 2022). Children and adolescents also presented with variations in physiological markers typically linked to the emergence of internalizing problems (e.g., Morales, Zeytinoglu, Buzzell, et al. 2022). Notably, children were acutely sensitive not only to the disruptions of their daily routines that came with school closings and diminished peer interactions, but also to broader societal or policy shifts. For example, 2‐ to 5‐year‐old children in an Israeli sample exhibited behavioral and emotional difficulties that ebbed and flowed with the imposition, relaxation, and then reimposition of lockdowns (Gordon‐Hacker et al. 2022). Together, emerging evidence suggests that the pandemic increased the likelihood that a child may experience internalizing symptoms as a function of both proximal and distal pandemic‐related disruptions to their daily life.

Despite converging results in later childhood and adolescence, studies assessing the pandemic's impact on infant outcomes have yielded mixed results, often reporting either a mild influence or no direct effects. For example, in a sample of infants born during the pandemic, López‐Morales et al. (2022) reported no direct association between maternal reported pandemic‐related negative experiences and infant negative affect. Instead, they found that maternal anxiety at the second and third trimester of pregnancy mediated the link between negative experiences due to the pandemic and infant negative affect. In another sample, infants born during the pandemic scored lower on maternal‐reported measures of fine motor, gross motor, and personal‐social skills at 6 months of age, relative to a historical cohort of infants born and assessed prior to the pandemic (Shuffrey et al. 2022). In contrast, Fiske and colleagues found no direct impact on temperament in a sample ranging from 6 to 48 months of age (Fiske et al. 2021).

There may be several reasons for these mixed results. For instance, it is possible that infants may have been indirectly impacted by COVID‐19 through pandemic‐related changes to family functioning (e.g., López‐Morales et al. 2022). Infants were not consciously aware of the turmoil driven by COVID‐19, and it is unlikely that they actively compared their experience to the pre‐pandemic period, an intrinsic comparison that may have contributed to mental health concerns seen in older children and adults. Moreover, caregivers may also have worked to buffer infants' immediate environments from pandemic‐related stressors, possibly sacrificing their own needs to fulfill their children's physical and socioemotional needs (Panchal et al. 2023; Roche et al. 2022).

Practically speaking, research into the effects of the pandemic on infant outcomes has relied mainly on a single assessment of caregiver‐reported temperament traits (e.g., MacNeill et al. 2023; Werchan et al. 2022) or has focused on child and maternal mental health after the pandemic onset (e.g., Buthmann et al. 2022; López‐Morales et al. 2022). These methodological constraints hinder our ability to detect unique effects of pandemic‐related changes on infant emotional development. Studies have also differed based on whether the sample consisted of infants born prior to or after the pandemic onset, further complicating the comparability of results. Some studies have used archival data to provide similar, but not identical, pre‐pandemic comparison groups (e.g., Shuffrey et al. 2022). Very few infant studies have data collected at multiple time points both before and after the pandemic onset.

Therefore, it is unlikely that studies examining the impact of COVID‐19 on infant outcomes will find unequivocal evidence of concurrent distress or dysfunction. Instead, functional differences may only become evident over time when caregivers and clinicians can compare emergent behaviors to what might be expected developmentally. To this end, the literature has already identified a strong foundation of risk markers and socioemotional profiles that are evident early in life, change over time, and increase the probability of later mental health concerns that could be used to start tracking emerging developmental trajectories.

### 1.2 Negative Affect as an Early Marker of Psychopathology Risk

Converging evidence from developmental and clinical psychology supports the link between early temperament traits and childhood psychopathology risk (Dollar and Calkins 2019; B. D. Ostlund et al. 2021; Fox et al. 2023). The constellation of emotions and behaviors that comprise an infant's temperament is thought to be modestly stable from early childhood onward. Trait expression in the first years, however, may be malleable to early experiences, which may include large‐scale and unexpected environmental stressors, such as natural disasters (e.g., Zhang et al. 2018). As a result, temperament research may provide a useful lens through which to understand the potential impact of COVID‐19 on socioemotional well‐being in early life. We focus specifically on infant negative affect (anger, fear, sadness) given its link to risk for internalizing problems in childhood (Fox et al. 2023).

Negative affect tends to increase over the course of infancy, often peaking in the second year of life (Dollar and Calkins 2019; LoBue et al. 2019). This trajectory may reflect increases in developmentally appropriate frustration and self‐reflective disappointment as infant ambitions to explore the environment clash with caregiver‐imposed limitations. Higher levels of negative affect in infancy, particularly when coupled with poor regulatory ability or familial risk for psychopathology (e.g., de Vente et al. 2020), may be linked to early emerging patterns of social reticence. This social reticence, in turn, is associated with the later emergence of elevated anxiety and depression in adolescence and young adulthood. Indeed, the temperament trait of behavioral inhibition is evident in toddlerhood, is associated with increased negativity in infancy and social reticence in childhood, and is the strongest individual difference predictor for social anxiety later in life (see Fox et al. 2023; B. Ostlund and Pérez‐Edgar 2023 for reviews).

Trajectories of infant negative affect often vary as a function of expectable early experiences as infants learn to navigate various interpersonal interactions (e.g., Gunther et al. 2023; Vallorani et al. 2023). Less is known about the impact of a pandemic, where the stressor is widespread, lingering, and unpredictable in intensity. Emerging data suggest that early temperamental profiles are indeed associated with socioemotional responses to the pandemic. For example, Morales, Zeytinoglu, Lorenzo, et al. (2022) found that, like the general population, young adults with a history of behavioral inhibition displayed initial spikes in general and social anxiety at the beginning of the pandemic in 2020. However, they failed to show the rapid decrease in symptoms evident among their non‐inhibited peers. This pattern was further modulated by coping strategies and prior markers of error‐monitoring (Morales, Zeytinoglu, Buzzell, et al. 2022). In another study with the same sample, Zeytinoglu et al. (2021) found that early patterns of worry, evident in childhood, helped account for these patterns, which in turn fueled dynamic interactions with parental worry (Lorenzo et al. 2021; see also Murray et al. 2023). Given that early temperament traits may be affected by potent environmental forces, and that the COVID‐19 pandemic profoundly changed the social context in which a young child develops, it follows that patterns of infant negative affect may be altered by the pandemic. This potentiation in negative affect is likely also impacted by known risk factors that place young children at higher risk for maladaptation, including caregiver psychopathology and neighborhood structural disadvantage (Nelson and Gabard‐Durnam 2020; Witherspoon et al. 2023).

### 1.3 Caregiver Anxiety and Neighborhood Disadvantage as Proximal and Distal Risk Factors

Unsurprisingly, factors that potentiate childhood psychopathology risk were exacerbated by daily life disruptions linked to the pandemic (Murray et al. 2023). Caregiver anxiety and depression are associated with increases in social withdrawal and anxiety in temperamentally at‐risk children (Kiel and Kalomiris 2019). In infancy, caregiver anxiety has been associated with potentiated levels of emotional reactivity at 6 months of age (Davis et al. 2004) and greater attention biases to threat among 4‐ to 24‐month‐old infants (Morales et al. 2017). In addition, greater unpredictability or chaos in the home environment is associated with increased social withdrawal and anxiety (Davis et al. 2019). Variability in caregiver behavior is also associated with fluctuations in infant negative affect (Gunther et al. 2023) and attention bias to threat (Vallorani et al. 2023).

Available data documenting an impact of COVID‐19 on infant functioning suggests that outcomes are mediated or moderated by the socioemotional concerns of caregivers. For example, in a sample of Italian families, infants' regulatory capacities at 3 months were indirectly related to prenatal stress experienced during the pandemic (Provenzi et al. 2023). That is, greater maternal anxiety was linked to increased parenting stress and reduced maternal bonding, both of which were associated with lower infant regulatory capacity. Similar patterns have been documented in U.S. samples, where maternal anxiety and pandemic‐related stress were linked to infant temperament and developmental outcomes (López‐Morales et al. 2022; MacNeill et al. 2023). Findings from older children further support these relationships, and studies from Brazil show increased distress and mental health concerns among children whose families experienced greater pandemic‐related stress (Murray et al. 2023), while positive parent‐child communication was protective against anxiety and depression (see systematic review: Panchal et al. 2023). Across contexts, the fluctuating nature of the pandemic triggered worry and uncertainty (Gordon‐Hacker et al. 2022; Murray et al. 2023) and made it more difficult for individuals and families to reach equilibrium—a “new normal.”

As with many social and economic disruptions, the weight of the pandemic was not evenly distributed across communities in the United States. Individuals from marginalized communities, often communities of color or individuals with lower socioeconomic resources, faced higher levels of COVID‐19 exposure, economic losses, disruptions to family life, and mental health concerns (Thomason et al. 2022). Many of these findings mirror known associations from the pre‐pandemic period, whereby neighborhood composition (e.g., poverty, unemployment, lower quality educational opportunities) predicts childhood mental and physical well‐being (Witherspoon et al. 2023). Given the impact of structural support on parental functioning and the downstream impact of parental distress on infants, we incorporated neighborhood structural disadvantage into our analyses for a comprehensive assessment of the pandemic's impact on infant emotional development.

### 1.4 Present Study

In this study, we examined whether trajectories of infant negative affect differed as a function of the COVID‐19 pandemic onset. We predicted that negative affect would increase linearly from 4 to 24 months of age, consistent with existing evidence (Dollar and Calkins 2019; LoBue et al. 2019). We then considered whether pandemic onset moderated these negative affect trajectories. In this case, we predicted that negative affect trajectories would increase (i.e., become steeper) post‐pandemic onset relative to the pre‐pandemic period. This hypothesis was informed by prior studies reporting a relative increase in internalizing symptoms among older children after the pandemic onset. Lastly, we explored whether caregiver anxiety influenced negative affect trajectories in the context of exposure to the COVID‐19 pandemic and existing environmental risk (neighborhood disadvantage).

## 2 Method

### 2.1 Participants

Participating infants were part of a larger longitudinal study (*N* = 357) that examined the relation between attention and (Pérez‐Edgar et al. 2021). Infants with at least one time‐point of temperament data were included in the present study (*N* = 330, 51% female). We recruited families from three research sites that ranged from rural to urban in the United States—Site 1‐State College, Pennsylvania (*n* = 166, 50% female), Site 2‐Harrisburg, Pennsylvania (*n* = 74, 45% female), and Site 3‐Newark, New Jersey (*n* = 90, 57% female). Caregivers consented for themselves and their infant to participate in each assessment, which occurred when their infant was approximately 4, 8, 12, 18, and 24 months of age.

Data were collected from October 2016 to August 2021, overlapping with the World Health Organization's designation of the COVID‐19 pandemic—March 11, 2020 (i.e., “pandemic onset”). All infants in this study were born prior to the pandemic onset. We therefore focused our analyses exclusively on postnatal experiences of the COVID‐19 pandemic. For reporting purposes only, Table 1 includes the sample's demographic information by whether data were available for the child before the onset of the pandemic only or if they provided at least one datum after the pandemic onset. Analyses did not use this person‐level categorical “binning.”

**TABLE 1:** Sample's description of their demographic variables.

Variables	Only pre‐onset data	Some post‐onset data
Data collection site		
Site 1 (State College, Pennsylvania)	117 (51.54%)	49 (47.57%)
Site 2 (Harrisburg, Pennsylvania)	51 (22.47%)	23 (22.33%)
Site 3 (Newark, New Jersey)	59 (25.99%)	31 (30.10%)
Total	227	103
Child's sex		
Male	117 (51.54%)	46 (44.66%)
Female	110 (48.46%)	57 (55.34%)
Child's race & ethnicity		
Asian/Pacific Islander	5 (2.20%)	4 (3.88%)
Hispanic	44 (19.38%)	22 (21.36%)
White, non‐Hispanic	125 (55.07%)	54 (52.43%)
African American, non‐Hispanic	32 (14.10%)	15 (14.56%)
Mixed	19 (8.37%)	8 (7.77%)
Did not disclose or missing	2 (0.88%)	NA
Family income		
$15,000 or less	35 (15.42%)	10 (9.71%)
$16,000–$20,000	15 (6.61%)	4 (3.88%)
$21,000–$30,000	13 (5.73%)	8 (7.77%)
$31,000–$40,000	11 (4.85%)	5 (4.85%)
$41,000–$50,000	17 (7.49%)	5 (4.85%)
$51,000–$60,000	24 (10.57%)	5 (4.85%)
Above $60,000	92 (40.53%)	48 (46.60%)
Did not disclose or missing	20 (8.81%)	18 (17.48%)
Mother's education		
Grade school or less	8 (3.52%)	1 (0.97%)
Some high school	12 (5.29%)	4 (3.88%)
High school graduate	22 (9.69%)	12 (11.65%)
Trade, technical, or some college	39 (17.18%)	17 (16.50%)
College graduate	56 (24.67%)	17 (16.50%)
Graduate training	39 (17.18%)	18 (17.48%)
Graduate degree	41 (18.06%)	25 (24.27%)
Did not disclose or missing	10 (4.41%)	9 (8.74%)

*Note:* Demographic information for participating families who completed data collection prior to the onset of the COVID‐19 pandemic (“Only Pre‐Onset Data”) and those who provided some data before and after the pandemic onset (“Some Post‐Onset Data”).

Instead, individual infant data points were categorized as pre‐ or post‐pandemic onset relative to the infant's age and the date when data were collected (PandemicStatus). We incorporated a dummy‐coded variable (covid_status) to indicate whether each data point was collected before or after the pandemic onset. The crucial aspect of our model is the interaction between child age and covid status. This interaction term enabled us to test whether the child's slope of negative affect differed in the post‐pandemic period compared to the pre‐pandemic period.

The present study was conducted according to guidelines laid down in the Declaration of Helsinki and in compliance with the ethical standards of the American Psychological Association, with written informed consent obtained from a parent or guardian for each child before any assessment or data collection. All procedures and materials in this study were approved by the Institutional Review Boards at Pennsylvania State University and Rutgers University, Newark. Detailed sample recruitment and study procedure information is reported elsewhere (Pérez‐Edgar et al. 2021).

Caregivers reported sociodemographic information for themselves and their infants at the initial assessment (Table 1). From the caregiver report, 47 infants were classified as African American, Non‐Hispanic (14%), nine infants were classified as Asian or Pacific Islander (3%), 66 infants were classified as Hispanic (20%), 27 infants were classified as Mixed race (8%), 179 infants were classified as White, Non‐Hispanic (54%), and two infants did not have race or ethnicity information reported (1%). A total of 26% of participating families reported their family income as $30,000 or less, while 42% reported their family income as $60,000 or greater.

### 2.2 Measures

#### 2.2.1 Infant Negative Affect

Mothers reported on their infant's temperament from 4 to 12 months of age using the Infant Behavior Questionnaire–Revised (IBQ‐R; Putnam et al. 2014). The IBQ‐R is a 191‐item questionnaire that assesses how frequently an infant displays certain behaviors in the past week. Items are scored on a 7‐point Likert scale that ranges from “Never” to “Always,” with an optional “Not applicable” response if a behavior was not observed in the past week. Items load onto one of 14 scales that then load onto three broad factors, including a Negative Affect factor. However, the three‐factor solution demonstrated poor fit within the full sample, with relatively low comparative fit indices (CFIs < 0.72) and high root mean square error of approximations (RMSEAs > 0.12) for models fit using 8 and 12‐month IBQ‐R data (see Zhou et al., under review, for details). This may be due, in part, to the racial, ethnic, and socioeconomic diversity of our sample relative to other studies that have utilized this measure (e.g., Gartstein and Hancock 2019). For this reason, we calculated a negative affect composite based on the Distress to Limitations, Fear, and Sadness subscales.

Mothers reported on their infant's temperament from 12 to 24 months of age using the Toddler Behavior Assessment Questionnaire (TBAQ; Goldsmith 1996). The TBAQ is a 120‐item questionnaire that assesses how frequently a young child displays specific behaviors in the past month. Items are scored on a 7‐point Likert scale that ranges from “Never” to “Always,” with an optional “Not applicable” response if a behavior was not observed in the past month. Items are loaded onto one of 11 scales. Scale scores are created by averaging items within each scale. We calculated a negative affect composite based on the Anger, Object Fear, Sadness, and Social Fear TBAQ scales to align with the IBQ‐R negative affect composite.

The expression of temperament traits changes over the first 24 months of life in conjunction with skill development in other domains (e.g., language, locomotion), despite presumed continuity in the underlying constructs themselves. To this end, we administered the IBQ‐R at the 4, 8, and 12‐month assessments and the TBAQ at the 12, 18, and 24‐month assessments. Scores on the IBQ‐R and TBAQ at 12 months of infant age, when parents completed both measures, were highly correlated (*r* = 0.70, *p* < 0.001; see Supporting Information S1). We chose to use the IBQ‐R data at 12 months in the present analyses.

#### 2.2.2 Maternal Anxiety

Mothers reported their own anxiety symptoms using the Beck Anxiety Inventory (BAI, Beck et al. 1988) at each time point of the study. The BAI is a 21‐item self‐report questionnaire that assesses cognitive (e.g., fear of losing control) and somatic (e.g., heart pounding/racing) anxiety symptoms. Items are scored on a 4‐point Likert scale that ranges from “Not at all” to “Severely.” Item scores are summed; higher scores indicate greater severity of anxiety symptoms. For our analyses, we split the time‐varying anxiety variable into “trait” and “state” components. In this case, “maternal trait anxiety” reflects how one person's average anxiety over the study period differs relative to the overall sample (i.e., sample‐mean centered, between‐person differences), while “maternal state anxiety” represents how a person's anxiety fluctuates over time relative to their own average anxiety (i.e., person‐mean centered, within‐person fluctuation). This centering approach reduces multicollinearity by separating distinct components of an individual's anxiety levels across the study period (Hoffman and Walters 2022; Kreft et al. 1995).

#### 2.2.3 Neighborhood Structural Disadvantage

A neighborhood disadvantage score was calculated for each family based on prior research into neighborhood dynamics and child development (Witherspoon et al. 2016). Family addresses at enrollment were used to obtain 2010 US census‐tract level data on five variables: *female‐headed households* (percent of female‐headed households in census tract), *unemployment* (percent of unemployed residents in the labor force), *educational attainment* (percent of individuals 25 years old or older without a high school diploma), *poverty level* (percent of residents whose income fell below the poverty level), and *family income* (average family income in census tract). These variables were standardized and averaged to create the neighborhood disadvantage score; higher scores indicate greater neighborhood disadvantage.

### 2.3 Analytic Plan

Analyses were conducted in R v4.2.2 (R Core Team 2022; see repository for data and analytic code to reproduce the analyses presented in this manuscript: https://osf.io/j3ey4/). Missing data were multiple imputed using the *mice* package (van Buuren and Groothuis‐Oudshoorn 2011). Multilevel models were estimated using the *lmer* package (Bates et al. 2015). Main effects and interactions were probed using the *interactions* (Long 2022) package.

Our predictor variables were:

- InfantAge: Infants' age in months (integer)
- MaternalAnxiety‐trait: Maternal Anxiety_*i*_ − Maternal Anxiety (overall average)
  ◦ Maternal Anxiety_i_ is the average anxiety level for mother *i* across all visits
- MaternalAnxiety‐state: Maternal Anxiety_it_ − MaternalAnxiety‐trait
  ◦ Maternal Anxiety_it_ is the anxiety level of mother *i* at time point *t*
- PandemicStatus: A dummy‐coded variable that represents when the specific data point was collected, where 0 is pre‐onset, and 1 is post‐onset
- NeighborhoodDisadvantage: Continuous variable created by averaging five census‐level variables that were first standardized

We considered negative affect at time point *t* for infant *i* as follows:

$${\text{NegativeAffect}}_{\text{it}}=\gamma_{00}+\gamma_{10}{\left(\text{InfantAge}\right)}_{it}+\gamma_{20}{\left(\text{MaternalAnxiety}‐\text{state}\right)}_{it}+\gamma_{01}{\left(\text{MaternalAnxiety}‐\text{trait}\right)}_{i}+\gamma_{02}{\left(\text{PandemicStatus}\right)}_{i}+\gamma_{03}{\left(\text{NeighbourhoodDisadvantage}\right)}_{i}+\gamma_{11}{\left(\text{InfantAge}\right)}_{it}{\left(\text{PandemicStatus}\right)}_{i}+\gamma_{21}{\left(\text{MaternalAnxiety}‐\text{state}\right)}_{it}{\left(\text{MaternalAnxiety}‐\text{trait}\right)}_{i}+\left[u_{0i}+u_{1i}{\left(\text{InfantAge}\right)}_{it}+u_{2i}{\left(\text{MaternalAnxiety}‐\text{state}\right)}_{it}+e_{it}\right],$$

where infant negative affect is modeled as a function of an intercept (γ_00_), two time‐varying covariates that represent the infant's age (γ_10_) and maternal state anxiety (γ_20_) at each assessment, and residual error (*e*_*ti*_). Parameters γ_01_, γ_02_, and γ_03_ reflect the main effects for maternal trait anxiety (sample mean‐centered), pandemic status for that data point (pre‐ or post‐pandemic onset), and neighborhood disadvantage on infant negative affect, respectively. The remaining two fixed effect parameters represent the cross‐level interactions between pandemic status and infant age (γ_11_) and between maternal trait and state anxiety (γ_21_). Random effects *u*_0*i*_ − *u*_2*i*_ reflect residual unexplained variation in respective intercepts and slopes.

Lastly, we compared the linear mixed effect model described above to a model that considered both linear and quadratic effects of time (i.e., infant age). The quadratic model, however, failed to converge. Therefore, we chose to use the model with only a linear effect of time in the main analyses.

## 3 Results

We present descriptive information on key variables in Table 2. Before the main analyses, we examined whether infants who completed data collection prior to the pandemic onset differed on key sociodemographic variables from those who provided one or more time points of data after the pandemic onset. The proportion of infants in each group did not differ by data collection site (χ ^2^(2, *N* = 330) = 0.66, *p* = 0.72), infant sex (χ ^2^(1, *N* = 330) = 1.08, *p* = 0.30), infant race/ethnicity (χ ^2^(4, *N* = 228) = 1.00, *p* = 0.91), family income (χ ^2^(6, *N* = 292) = 6.72, *p* = 0.35), or maternal education (χ ^2^(6, *N* = 311) = 5.73, *p* = 0.45). Moreover, infants did not differ across groups in the negative affect composite at the 4‐month time point (*t*(264) = 0.10, *p* = 0.92), which occurred prior to pandemic onset for all participants.

**TABLE 2:** Descriptive information on key variables.

	Mean (SD)	Range
Data collected^a^		
Prior to pandemic onset (“PreOnset”)	*n* = 867	
After pandemic onset (“PostOnset”)	*n* = 201	
Infant negative affect (IBQ‐R/TBAQ)^b^	3.23 (0.63)	[1, 5.30]
Maternal anxiety (BAI)^b^	6.37 (6.01)	[0, 44.33]
Neighborhood disadvantage composite (*z*‐scored)	0.07 (0.80)	[−1.39, 3.51]

^a^ Count of valid data points based on whether participants' involvement for that time point occurred before or after the onset of the COVID‐19 pandemic.

^b^ Calculated using each child's average scores across multiple time points.

In calculating the intraclass coefficient, we found that 52% of the total variance in negative affect was attributable to differences between infants, while the remaining 48% was due to variation within infants over time. This result supported the inclusion of both time‐varying and time‐invariant predictors to explain variability in negative affect. There was no evidence (*b* = −6.12e‐04, 95% CI [−1.68e‐03, 4.61e‐04], *t*(1053) = −1.12, *p* = 0.263, Std. *b* = −0.02) that the inclusion of the interaction terms between maternal anxiety as a trait and as a state was associated with children's negative affect scores. Thus, we performed another model comparison without this interaction, as seen below:

$${\text{NegativeAffect}}_{\text{it}}=\gamma_{00}+\gamma_{10}{\left(\text{InfantAge}\right)}_{\text{it}}+\gamma_{20}{\left(\text{MaternalAnxiety}‐\text{state}\right)}_{\text{it}}+\gamma_{01}{\left(\text{MaternalAnxiety}‐\text{trait}\right)}_{i}+\gamma_{02}{\left(\text{PandemicStatus}\right)}_{i}+\gamma_{03}{\left(\text{NeighborhoodDisadvantage}\right)}_{i}+\gamma_{11}{\left(\text{InfantAge}\right)}_{\text{it}}{\left(\text{PandemicStatus}\right)}_{i}+\left[u_{0i}+u_{1i}{\left(\text{InfantAge}\right)}_{\text{it}}+u_{2i}{\left(\text{MaternalAnxiety}‐\text{state}\right)}_{\text{it}}+e_{\text{it}}\right].$$

**FIGURE 1:**
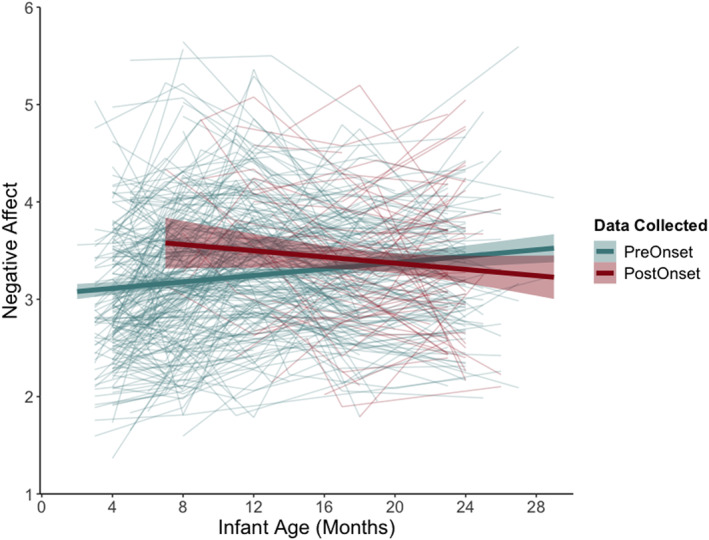
Infant negative affect trajectories. Infant negative affect trajectories prior to (“PreOnset,” green) and after (“PostOnset,” red) onset of the COVID‐19 pandemic. Bold lines with confidence intervals reflect the average for each condition, while thin lines depict each infant's negative affect trajectory.

We present findings from the final multilevel model in Table 3. The main effect of time was qualified by a significant interaction between infant age and pandemic status (*b* = −0.04, SE = 0.01, *p* < 0.001), such that the slope of negative affect decreased after the pandemic onset relative to the pre‐pandemic period. Simple slopes analysis revealed that the slope of negative affect was significant before and after the pandemic onset, although the effects were in opposite directions (Figure 1). That is, infant negative affect increased prior to pandemic onset (*b* = 0.02, *p* < 0.01) and decreased post‐pandemic onset (*b* = −0.02, *p* = 0.01).

**TABLE 3:** Multilevel model results.

Predictors	Negative affect			
	Standardized coefficients	CI	df	*p*
Intercept	3.04	[2.95–3.13]	344.59	**< 0.001**
Infant age (months)	0.02	[0.01–0.02]	291.87	**< 0.001**
Pandemic status	0.62	[0.31–0.92]	766.31	**< 0.001**
Neighborhood disadvantage	0.18	[0.10–0.26]	336.08	**< 0.001**
Maternal anxiety–trait (centered)	0.01	[0.00–0.02]	309.20	**0.009**
Maternal anxiety–state	0.00	[−0.01 to 0.01]	63.93	0.781
Infant age × pandemic status	−0.04	[−0.05 to −0.02]	810.65	**< 0.001**
Random effects				
σ ^2^	0.20			
τ_00 recrod_id_	0.34			
τ_11 record_id.infant_age_	0.00			
τ_11 record_id.maternal_anx_state_	0.00			
ρ_01_	−0.53			
	−0.49			
ICC	0.58			
*N*_record_id_	330			
Observations	1068			
Marginal *R*2/conditional *R*2	0.08/0.61			

*Note:* Bolded *p*‐values indicate statistical significance at the *p* < 0.01 level.

We also found that higher levels of maternal trait anxiety were related to higher levels of infant negative affect (*b* = 0.02, SE = 0.01, *p* = 0.002) regardless of pandemic timing. Maternal state anxiety did not predict infant negative affect (*p* < 0.655). Lastly, we found that neighborhood composition was related to negative affect (*b* = 0.18, SE = 0.04, *p* < 0.001), such that infants from neighborhoods with more structural disadvantage were reported to show higher levels of negative affect. All results were essentially the same when 15 outlier cases (Cook's distance ≥ 0.90) were removed.

## 4 Discussion

In the current study, we examined trajectories of negative affect within a relatively large and diverse cohort of infants, all recruited prior to the onset of the COVID‐19 pandemic. The unexpected emergence of a global pandemic interrupted normative, expectable environments for some of these infants at varying times across the first 2 years of life. This natural experiment provided a novel opportunity to examine how historical events can impact developmental systems, as laid out by Bronfenbrenner and other theorists (Pérez‐Edgar 2021).

The emerging literature has captured early effects of the pandemic on mental health, coping, and well‐being that are profound among children, adolescents, and adults (Kauhanen et al. 2023; Morales, Zeytinoglu, Lorenzo, et al. 2022; Murray et al. 2023; Panchal et al. 2023; Werchan et al. 2022; Xiong et al. 2020). However, questions remain regarding how long‐lasting these effects will be. In contrast, the literature focused on infants has been mixed (e.g., Fiske et al. 2021; Provenzi et al. 2023). Much of the infant work has focused on *in‐utero* exposure to either COVID‐19 infection or the stressors generated by COVID‐19 health risks and mitigation efforts (López‐Morales et al. 2022; MacNeill et al. 2023; Provenzi et al. 2023; Werchan et al. 2023). Thus, samples are relatively young and have few available time points—infants must literally grow into the developmental trajectories of interest. The prenatal and perinatal cohorts are, by definition, derived post‐pandemic, so it is difficult to disentangle the specific impact of the pandemic on development. The current study adds to this literature by examining a single cohort of infants, all born prior to the onset of the COVID‐19 pandemic. Although this precludes our ability to explore the potential impact of fetal programming components of the Developmental Origins of Health and Disease (DOHaD) model (B. Ostlund and Pérez‐Edgar 2023; Padmanabhan et al. 2016), the study does allow us to directly compare pre‐pandemic trajectories with pandemic‐interrupted developmental patterns.

### 4.1 Current Findings and Implications

Consistent with prior studies (Dollar and Calkins 2019; LoBue et al. 2019), we found that negative affect increased from 4 to 24 months of age pre‐pandemic. This rise may parallel the general increase in daily encounters that evoke frustration or disappointment across infancy, whereby an infant's ambition to explore is often at odds with caregiver‐imposed limitations. We then examined trajectories of negative affect following onset of the COVID‐19 pandemic, using data from infants who experienced the pandemic onset at varying points in the first 2 years of life. Contrary to our prediction, we found that negative affect *decreased* following the pandemic onset. We hypothesized that, given emerging evidence linking childhood mental health concerns and the COVID‐19 pandemic (e.g., Murray et al. 2023), negative affect would increase post‐pandemic onset at an increased rate relative to the expected developmental trajectory. The assumption was that the disruptions generated by the pandemic, including caregiver stress and generalized strain on family systems, would cascade to impact the infant. This prediction was based on strong and robust literature on the impact of caregiver distress, familial disruption, and socioeconomic strain on childhood socioemotional development (Nelson and Gabard‐Durnam 2020).

Nevertheless, early findings on the COVID‐19 pandemic's impact on infants should have tempered these expectations; that is, data to date are decidedly mixed. Studies have found few direct associations with negative affect or temperament among infants (Shuffrey et al. 2022), as effects were carried by caregiver functioning (Buthmann et al. 2022; Werchan et al. 2022). While some studies found impacts on neurodevelopment (e.g., Shuffrey et al. 2022), it is not clear how long‐lasting these effects may be. Conversely, other studies have found enhanced abilities among infants experiencing a pandemic‐mitigated environment. For example, pre‐pandemic infants had lower vagal tone during triadic interactions with their mothers and fathers versus post‐pandemic infants (Rattaz et al. 2023). Thus, infants who likely experienced more time with caregivers as mitigation efforts had families together at home may have had more opportunity to learn to regulate within the family system. Again, it remains to be seen how long‐lasting these differences may be.

Studies have found that impacts on infants, both positive and negative, are typically channeled through caregiver traits and behaviors or broader structural forces. For example, infants with caregivers demonstrating maladaptive coping strategies or increased internalizing symptoms during the pandemic tended to show disruptions in attentional processing (Werchan et al. 2023) and increased negative affect (López‐Morales et al. 2022). Indeed, worry regarding the impact of maternal COVID‐19 infection may have as much or greater impact on infant functioning than maternal infection status (Werchan et al. 2023). In contrast, caregivers who were able to call on social support networks, whether in person or virtually (Buthmann et al. 2022; Roche et al. 2022), showed better adaptation. At a societal level, infants in families and communities that are historically marginalized had fewer economic or social supports or were hardest hit by infection‐linked morbidity and mortality saw far greater disruptions to daily life (Thomason et al. 2022). It may be that greater care and monitoring will be needed for these infants going forward.

Within the scope of the current study, we speculate on reasons why we observed decreased negative affect after the onset of the COVID‐19 pandemic. First, compensatory processes may have been actively and systematically in place to help buttress infant impacts. As noted above, caregivers often drew on personal, familial, or community resources to buffer the daily experience of infants and children, providing consistent emotional support and stability during these challenging times.

Second, for the current sample, the expected environmental triggers for elevated negative affect may have eased during the COVID‐19 pandemic. Frustrated or blocked goals are a potential source of negative affect that, when paired with less effective self‐regulation abilities, may allow unchecked emotional reactivity to flourish (Dollar and Calkins 2019; B. D. Ostlund et al. 2021). With closed schools and work‐from‐home requirements, infants may have spent relatively more time in direct one‐on‐one interactions with caregivers. This contact, coupled with fewer competing interests, may have provided fewer triggers for negative affect and allowed for swifter external regulatory interventions from caregivers. Thus, caregivers were reporting less expression of negative affect because there was less opportunity for negative affect.

Third, and at the extreme end of the spectrum relative to the prior two possible explanations, it may be that prolonged or fluctuating levels of pandemic‐related chaos and uncertainty contributed to a blunted pattern of negative affect modulation (e.g., Raver et al. 2015). Indeed, prior work (Gunther et al. 2023) has found that greater fluctuation over time in maternal attention biases to threat is related to decreases in infant negative affect over time. At the same time, the fluctuating circumstances surrounding mitigation efforts may have limited infants' exposure to developmentally appropriate challenges that typically evoke negative affect (e.g., interactions with adults outside the close familial unit), potentially hindering opportunities to practice more mature emotion regulation skills. In this case, expected increases in negative affect may emerge later in early childhood, when the young child is introduced to social contexts (e.g., preschool) that challenge potentially less developed regulatory capacities. These speculative scenarios will require follow‐up work to disentangle.

### 4.2 Limitations

The current study should be assessed in light of its limitations. First, COVID‐19 mitigation required that we shut down in‐person data collection to help ease disease transmission. As such, infants who completed the study prior to onset of the COVID‐19 pandemic had complete data, including direct behavioral observation of temperament and parent‐child interactions, eye‐tracking, electroencephalography, and respiratory sinus arrhythmia (Pérez‐Edgar et al. 2021). These data were lost for the infants whose visits were interrupted by the COVID‐19 pandemic. As such, any analyses comparing trajectories must depend on data that could be collected virtually. In this case, this meant caregiver‐reported temperamental negative affect. There is some concern regarding biased reporting due to caregiver traits and experiences that could not be thoroughly compared to observed profiles. However, recent work suggests that caregivers can provide unbiased reports despite variations in their own personality traits or mental health symptoms (Olino et al. 2021). In addition, other known effects associated with maternal anxiety (self‐report) and neighborhood disadvantage (external data) reflected our initial assumptions, suggesting a unique association with the pandemic.

Second, while the sample is relatively large and diverse, it does not capture the full spectrum of communities impacted by the COVID‐19 pandemic. Even with the range in geography, income, and parental backgrounds, the sample is relatively well‐off and was likely insulated from the most extreme direct consequences of the pandemic. Moreover, we had insufficient data that could speak to COVID‐19‐specific hardships endured by the participating families. Like much of the literature, we therefore cannot speak to the experiences of infants facing dire economic hardship, community upheaval, or even parental loss.

Third, we do not have direct measurements of the mechanism that could have led to the differences across the participants. Our natural experiment allowed us to isolate shifts that emerged coincident with the emergence of the COVID‐19 pandemic. However, we cannot say with certainty *why* the trajectories emerged as they did. Future work will be needed to tease apart potential mechanisms that either varied with the pandemic onset or placed some infants at greater risk given the pandemic onset.

Fourth, we cannot speak to what long‐term consequences, if any, may emerge from the shift in negative affect trajectories noted here. Prior work has shown that under normative circumstances, increased negative affect, if high and stable over time, is associated with social withdrawal, peer difficulties, and social anxiety (Fox et al. 2023). In extreme circumstances of long‐term neglect (Cicchetti and Valentino 2015) or even institutionalization (Moulson et al. 2015) blunted negative affect is a marker of later risk. Follow‐up longitudinal studies will be needed to ascertain where our currently observed patterns land along this spectrum.

### 4.3 Future Directions and Conclusion

Even with these caveats, the current study adds to a growing body of literature examining the impact of a global pandemic on the lives of the youngest among us. Many of these studies have begun following the pandemic onset, often examining the effects of *in utero* exposure. Other studies have leveraged longitudinal studies to examine how pre‐pandemic functioning can help predict post‐pandemic profiles. The current study adds to these diverse approaches by examining pre‐ and post‐pandemic trajectories within a single sample of infants. Here, we found that in contrast to expectations, trajectories of negative affect decreased post‐pandemic onset. Additional work is needed to understand better the mechanisms fueling this trajectory. In addition, we need more time to see how, or if, the early impact of the COVID‐19 pandemic has long‐term consequences for development. The COVID‐19 pandemic has been singular in its global, profound, and lingering impact on the health and well‐being of individuals and families. It is unlikely that all effects are acute and time‐limited. Instead, we know that early experiences can have profound impacts on development that only emerge as the systems of interest mature or are stressed by developmentally‐typical challenges (Padmanabhan et al. 2016; B. Ostlund and Pérez‐Edgar 2023; Pérez‐Edgar 2021). Work such as the COVID‐19 Perinatal Experiences (COPE) project (Buthmann et al. 2022) and the Covid Generation (COVGEN) alliance (Werchan et al. 2022) may help better understand patterns of both risk and resilience across the subsequent decades of life.

## Author Contributions

**Joscelin Rocha‐Hidalgo:** data curation, formal analysis, validation, visualization, writing – review and editing. **Brendan Ostlund:** conceptualization, data curation, formal analysis, funding acquisition, validation, visualization, writing – original draft, writing – review and editing. **Vanessa LoBue:** conceptualization, funding acquisition, project administration, writing – review and editing. **Kristin A. Buss:** conceptualization, funding acquisition, project administration, writing – review and editing. **Koraly E. Pérez‐Edgar:** conceptualization, funding acquisition, project administration, supervision, writing – original draft, writing – review and editing.

## Ethics Statement

The present study was conducted according to guidelines laid down in the Declaration of Helsinki and in compliance with the ethical standards of the American Psychological Association, with written informed consent obtained from a parent or guardian for each child before any assessment or data collection. All procedures and materials in this study were approved by the Institutional Review Boards at Pennsylvania State University and Rutgers University, Newark.

## Supporting information


**Figure S1**. Correlation between negative affect measures at 12 months of age.

## Data Availability

The analyses presented here were not preregistered. The analytic code and data necessary to reproduce the analyses presented in this manuscript are publicly accessible at the following URL: https://osf.io/j3ey4/.
