# Supplementary material for: When the World Pivots: Changes in Infant Negative Affect Trajectories Following the Onset of the COVID‐19 Pandemic
Source: Infancy. 2025 Aug 6;30(4):e70041. doi: 10.1111/infa.70041 (PMC12329392; doi:10.1111/infa.70041)

**Supplementary Materials**

**S1. Correlation between negative affect measures at 12 months of age**

Scores on the IBQ-R and TBAQ at 12 months of infant age, when parents completed both

measures, were highly correlated (*r* = .70, *p* <.001).


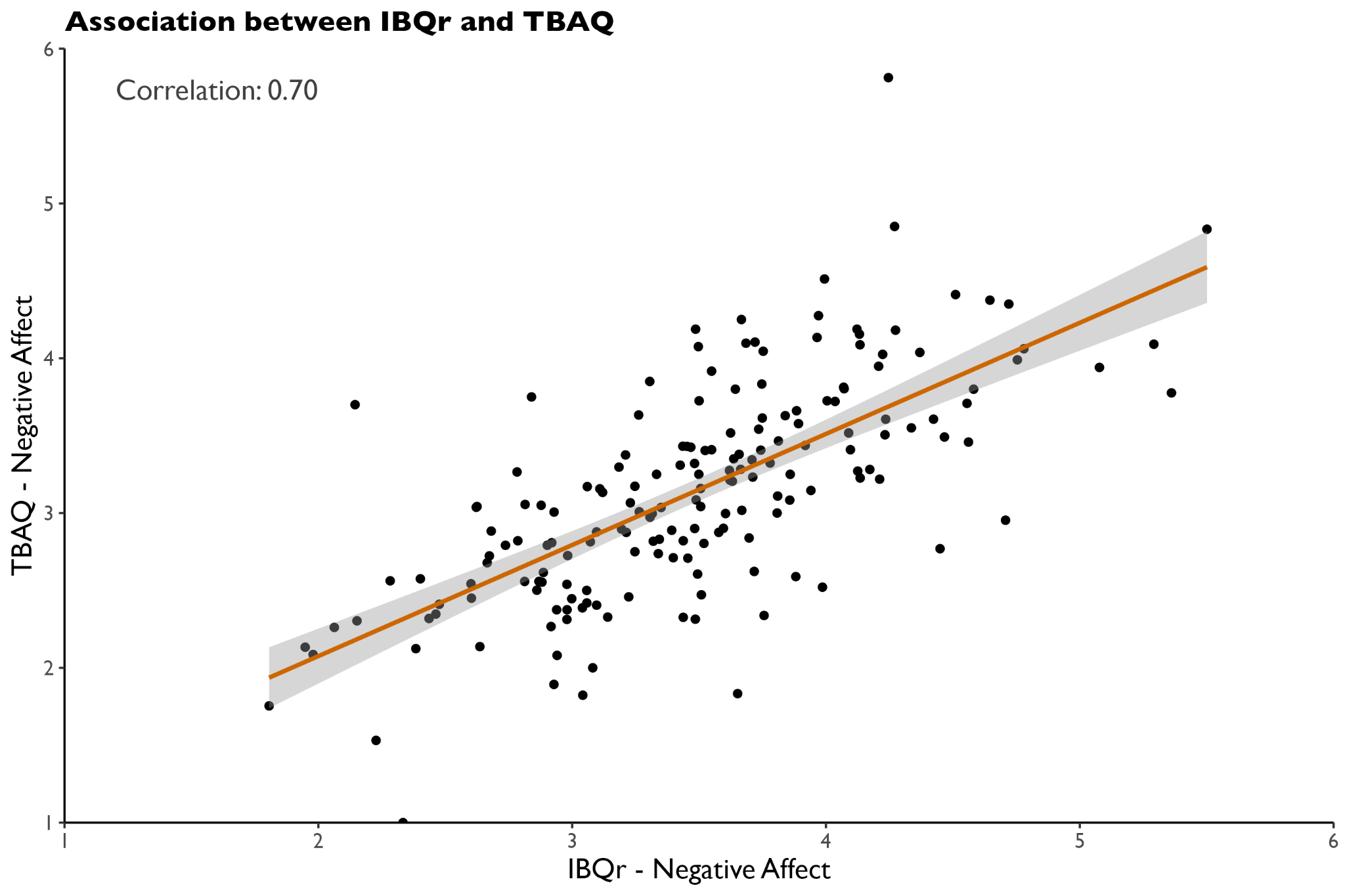

Supplement: Supplementary file 1 — Figure S1. Correlation between negative affect measures at 12 months of age. [file INFA-30-0-s001.docx]
